# Neuromodulatory Focused Ultrasound for Epilepsy: Are Animal Models Useful?

**DOI:** 10.1021/acschemneuro.4c00198

**Published:** 2024-04-18

**Authors:** Po-Chun Chu, Hsiang-Yu Yu, Robert S. Fisher, Hao-Li Liu

**Affiliations:** a Department of Electrical Engineering, National Taiwan University, Taipei City 10617, Taiwan; b Department of Neurology, Taipei Veteran General Hospital, Taipei City 11217, Taiwan; c School of Medicine, National Yang Ming Chiao Tung University, Taipei City 112304, Taiwan; d Department of Neurology and Neurological Sciences, Stanford University School of Medicine, Palo Alto, California 94305, United States

**Keywords:** Focused ultrasound, neuromodulation, epilepsy, kainic acid model, clinical trial, translation

## Abstract

Ultrasound neuromodulation is a potential alternative therapy for suppressing epileptic discharges. Recently, several human clinical trials have reported promising results from repeated focused ultrasound (FUS) treatments for temporal lobe epilepsy. In this Viewpoint, we highlight the valuable guidance of preclinical validation methods for choosing the optimal FUS parameters, thus ensuring consistency with the outcomes of clinical trials and leading human trials to the safest and most effective approaches.

Epilepsy affects approximately 50 individuals per 100,000 annually.^1,2^ While antiseizure medications form the cornerstone of epilepsy therapy, drug-resistant epilepsy (DRE) is observed in 20–40% of patients.^1,3^ Patients with DRE who are not good candidates for resective or ablative surgery may undergo neuromodulation therapy, encompassing responsive neurostimulation (RNS), deep brain stimulation (DBS), or vagus nerve stimulation (VNS). While these interventions provide significant palliative benefits, their application is limited, and many patients may not meet the criteria for eligibility.^2^

Transcranial focused ultrasound (FUS) is a cutting-edge technology with the capability to precisely target deep brain tissue noninvasively while minimizing impact on adjacent regions.^4^ Recent studies have revealed that employing FUS to induce neuromodulatory effects holds promise in suppressing epileptiform discharges, thus offering potential for treating epilepsy.^2^ Several human clinical trials employing repeated FUS treatments for temporal lobe epilepsy (TLE) have been conducted, revealing promising therapeutic potential (see details in Supplementary Table 1). In 2021, Lee *et al.* conducted a pilot study on FUS for epilepsy at the Taipei Veterans General Hospital (VGH), Taiwan, reporting a reduction in seizures within 72 h in 1/3 of the patients.^1^ Stern *et al.* at the University of California, Los Angeles (UCLA), USA, utilized excitatory and inhibitory stimulation paradigms to examine the safety of FUS on TLE patients, noting no significant histopathologic damage in brain tissue.^4^ Moreover, a recent human pilot safety trial reported by Bubrick *et al.* at Brigham and Women’s Hospital (BWH) employed serial FUS treatments targeting the hippocampus in TLE, demonstrating an average 50% reduction in seizures by the end of the 6-month follow-up phase.^3^

The swift progression of the clinical trial translation involving the use of FUS for epilepsy owes its momentum in part to the success of preclinical validation. Chronic epilepsy animal models have consistently demonstrated that FUS session effectively suppresses epileptic signals in kainic acid (KA) models for up to seven weeks.^2^ Duration of any therapeutic effect is of additional importance. Patients would not be likely to submit to daily sonications for an indefinite period of time. A recent BWH clinical trial used a design of six successive sonications to DRE patients within three weeks.^3^ Our second study of FUS effect on kainate-induced seizures administered sonication in two sessions separated by five weeks. This extension resulted in notable reduced hippocampal inflammation and an improvement in behavioral problems of epilepsy.^5^ With the experimental groups included in the study design, a total of 33 animals underwent electrode implantation and underwent sonication to assess burst suppression (refer to Figure 1A), allowing for the replication of parameters from all four currently existing clinical trials.

**Figure 1:**
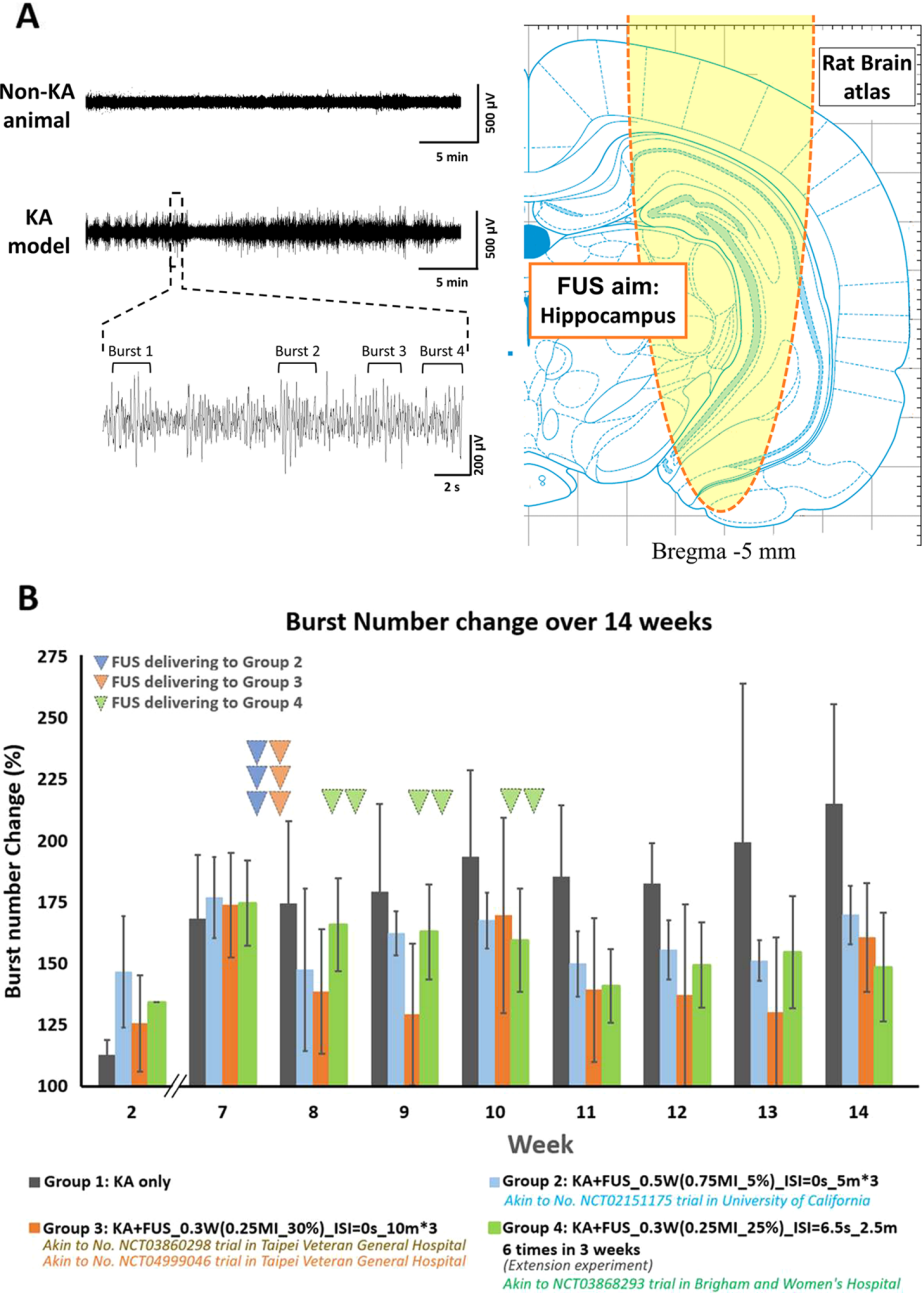
(A) EEG recordings in KA models and KA models that receive FUS treatment (KA + FUS). FUS targeted the hippocampal region in the KA model. (B) The change of epileptic burst counts was recorded for 14 weeks and the FUS parameters akin to clinical trials and extension experiments are marked with colors and italic type. KA was administered at week 1–2, and the epileptiform discharges were stably presented after week 7.

The clinical trial at UCLA (NCT02151175) used a single-element MR-compatible FUS transducer to target hippocampal regions, with the sonication parameters of mechanical index (MI) of 0.75–2.14, pulse repetition frequency (PRF) of 500 Hz, 5% duty cycle (DC), and peak-spatial-time-averaged intensities (*I*_SPTA_) of 0.72–5.8 W/cm^2^.^4^ Later, the clinical trial at VGH was conducted (NCT03860298) with the parameters of 0.25 MI, 100 Hz PRF, 30% DC to target the onset sites identified by the stereotactic EEGs.^1^ Recently, the study done at the BWH (NCT03868293) delivered sonication with the parameters of two days a week for 3 weeks, with a 0.19–0.57 MI, PRF of 500 Hz, 18.3% DC and *I*_SPTA_ of 0.5–1.1 W/cm^2^.^3^

We utilized the KA-induced epilepsy model to mimic the ongoing clinical trials: group 1, KA intra-amygdala models; group 2, KA animals subjected to 500 kHz FUS, with 0.75 MI, 5% DC (to mimic ultrasound parameters in the NCT02151175 trial^4^). Group 3 of KA models received three 10 min durations of 0.25-MI FUS, DC of 30% with *I*_SPTA_ of 0.3 W/cm^2^ (to mimic ultrasound parameters in the NCT03860298 trial^1^ and provide hints for the ongoing NCT04999046 trial). In group 4, a 6.5 s interstimulus interval (ISI) was implemented in sonication session and involved KA models that received six sonication sessions (*I*_SPTA_ = 0.3 W/cm^2^, ISI = 6.5 s, duration = 2.5 min) throughout weeks 7–9 (to mimic ultrasound parameters in the NCT03868293 trial^3^).

Figure 1B summarizes the numbers of epileptic bursts among groups. The KA-only cohort (group 1) was as the control group and exhibited a stable epileptiform discharge increase of 168.4% after week 7. In group 3, three 10-min 0.25 MI sonications with 30% duty cycle reduced epileptic bursts to 138.7% at week 8, sustaining significance until week 14 (*p* < 0.05 in 6 weeks). In group 2, decreasing DC and duration of FUS led to less significant inhibitions (*p* < 0.05 in 4 weeks for group 2). Sonication with ISI displayed the least significant inhibitions (group 4). However, increasing delivery frequency of low dosing FUS exposure (*I*_SPTA_ = 0.3 W/cm^2^, delivered 6 times in three weeks) still exhibited an accumulation of burst suppression effect (*p* < 0.05 in 3 weeks).

The seizure suppression effects observed in group 2 were less pronounced compared to the other two groups, consistent with the clinical findings reported in their respective trial documents.^4^ Conversely, parameters in group 3 exhibited a clear seizure suppression effect. Thus, based on this promising preclinical evidence, the parameters utilized in group 3 were selected for the phase II clinical trials conducted in VGH (NCT04999046, currently recruiting). Moreover, efficacy of FUS treatment design found at the BWH was also similar to the burst change in group 4 after receiving sonication two days a week for 3 weeks.^3^ This underscores the potential of animal models of epilepsy in guiding the selection of optimal FUS parameter settings, despite substantial differences between rat and human brains. This suggests that animal models of epilepsy may have value for choosing among the very large numbers of possible FUS parameter settings, despite vast differences in rat and human brain.

Animal experiments can provide evidence about safety. Our previous experiments with the KA model not only demonstrated safe parameters but showed histological preservation by FUS, compared to KA-only animals. Nevertheless, we did identify high intensities of FUS that can induce tissue damage.^2^ The BWH and UCLA trials did not demonstrate MRI or pathological (after resection) tissue injury, again concordant with our animal data. Animal models have also offered invaluable insights into the potential mechanisms underlying FUS neuromodulation in epilepsy. Several studies have shown that FUS can transiently modulate transmembrane sodium or calcium channels and may also induce long-term neuroprotective effects by reducing inflammation and gliosis in brain tissue.^2,5^ Moreover, in preclinical animal experiments, we identified that FUS also protects brain tissue and partially preserves normal behavior.

Current preclinical or clinical findings both suggest that the FUS has the potential to long-term suppress epileptiform EEG activity with a wide range of FUS parameter selections. Controlled experiments will be needed to document clinical safety and to quantify efficacy. Long-term efficacy of invasive neuromodulation approximates 50–80% for DBS, VNS, and RNS (details listed in Supplementary Table 2). The question of whether FUS will match the effectiveness of invasive neuromodulation requires further investigation. However, FUS offers distinct advantages, including its deep, precise targeting and, most notably, its noninvasive nature. Findings originated from the preclinical testing should be able to gain the understanding and pave the way for exploring potential therapeutic approach for epilepsy treatment.

FUS therapy for epilepsy is a very new field, and information is rapidly accumulating. Experience to date suggests great promise. Experiments in animal models will help to guide human trials toward the safest and most effective approaches.

## Supporting Information Available

Supplementary Table 1 listing comparative overview of clinical trials utilizing low-intensity FUS for Epilepsy (PDF)
Supplementary Table 2 listing comparison of efficacy of inhibition of seizure frequency among neuromodulation tools (PDF)
