# Supplementary material for: Neuromodulatory Focused Ultrasound for Epilepsy: Are Animal Models Useful?
Source: ACS Chem Neurosci. 2024 Apr 18;15(9):1728–31. doi: 10.1021/acschemneuro.4c00198 (PMC11066834; doi:10.1021/acschemneuro.4c00198)
Supplement: Supplementary file 1 — cn4c00198_si_001.pdf [file cn4c00198_si_001.pdf]

Supplementary Table 1. Comparative overview of clinical trials utilizing low-intensity FUS for Epilepsy. I_{SPTA}= Spatial-peak temporal-averaged intensity; ISI = inter-stimulus interval; MI = mechanical index; f_0 = center frequency. PRF = pulse repetition frequency. [a] Details in NCT03860298 trial; [b] Details in NCT04999046 trial

**Clinical trial no.**	**NCT02151175**	**NCT03868293**	**NCT03860298[a]**<br>**NCT04999046[b]**
Site Location	*University of California, Los Angeles, USA*	*Brigham and Women's Hospital, Boston, USA*	*Taipei Veteran General Hospital, Taipei, Taiwan*
**Device,**<br>$\boldsymbol{f_0}$	LIFUP<br>650 kHz	PLIFUS<br>548 kHz	NaviFUS system<br>500 kHz
**MPa,**<br>**MI**	0.61 - 1.72 MPa<br>0.75 - 2.14 MI	0.14 - 0.42 MPa<br>0.19 - 0.57 MI	[a] 0.54 MPa/ 0.75MI<br>[b] 0.18 MPa/ 0.25 MI
**PRF, Duty**	100 Hz, 5%	500 Hz, 18.3 %	100 Hz, 30%
**I_{SPTA}, target**	0.72–5.8 W/cm^2	0.50–1.1 W/cm^2	[a] 2.8 W/cm^2<br>[b] 0.3 W/cm^2
**ISI**	NA	6.5 s	NA
**Duration**<br>**Deliver strategy**	30s<br>2 sonication in one day	140s for each target<br>6 sonications in 3 weeks	[a] 600s, 1 sonication in one day<br>[b] 300s, 3 sonication in one day
**Target**	Anterior temporal lobe	Hippocampus	Seizure onset zone
**Primary finding**	No significant histopathologic damage	Seizure reduction (average ~ 50 %)	[a] Decrease in seizure frequency within 72 h in 1/3 patients<br>[b] Ongoing
Year of publishing	*2021*	*2024*	*[a] 2021*<br>*[b] unpublished data*
