# Supplementary material for: Neuromodulatory Focused Ultrasound for Epilepsy: Are Animal Models Useful?
Source: ACS Chem Neurosci. 2024 Apr 18;15(9):1728–31. doi: 10.1021/acschemneuro.4c00198 (PMC11066834; doi:10.1021/acschemneuro.4c00198)
Supplement: Supplementary file 2 — cn4c00198_si_002.pdf [file cn4c00198_si_002.pdf]

Supplementary Table 2. Comparison of efficacy of inhibition of seizure frequency among neuromodulation tools

**Neuromodulation tool**	**Ultrasound**	**Deep Brain Stimulation (DBS)**	**Vagal nerve stimulation (VNS)**	**Responsive NeuroStimulation (RNS)**
**Modulating site**	Hippocampus	Anterior nucleus thalamus	Vagal nerve	Cortex
**Responding rate**	83 %	74%	50%	84%
**Short-term outcome (within 1 year)**	**6 months:** [a]50%	**4 months:** [b]40.4%	**3 months:** [c]31%	**5 months:** [d]38%
**Long-term outcome (More than 3 year)**	N.A.	**7 years:** [e]75%	**12 Years:** [f]53%	**≥ 3 years:** [g]74%

a. Bubrick *et al.* Transcranial ultrasound neuromodulation for epilepsy: A pilot safety trial. Brain Stimulation: Basic, Translational, and Clinical Research in Neuromodulation. Brain Stimulation. 2024.

b. Fisher *et al.* Electrical stimulation of the anterior nucleus of thalamus for treatment of refractory epilepsy. Epilepsia. 2010

c. Ben-Menachem *et al.* Vagus nerve stimulation for treatment of partial seizures: 1. A controlled study of effect on seizures. First International Vagus Nerve Stimulation Study Group. Epilepsia. 1994.

d. Morrell *et al.* Responsive cortical stimulation for the treatment of medically intractable partial epilepsy. Neurology. 2011.

e. Salanova *et al.* The SANTÉ study at 10 years of follow-up: effectiveness, safety, and sudden unexpected death in epilepsy. Epilepsia. 2021.

f. Uthman *et al.* Effectiveness of vagus nerve stimulation in epilepsy patients: a 12-year observation. Neurology. 2004.

g. Razavi B *et al.* Real-world experience with direct brain-responsive neurostimulation for focal onset seizures. Epilepsia. 2020.
